# LC-MS/MS-Based Determination and Optimization of Linoleic Acid Oxides in *Baijiu* and Their Variation with Storage Time

**DOI:** 10.3390/metabo15040246

**Published:** 2025-04-02

**Authors:** Cheng Fang, Xiaotong Zhuang, Zhanguo Li, Yongfang Zou, Jizhou Pu, Dong Wang, Yan Xu

**Affiliations:** 1 Laboratory of Brewing Microbiology and Applied Enzymology, School of Biotechnology, Jiangnan University, Wuxi 214122, China; cfang@jiangnan.edu.cn (C.F.); 6220210042@stu.jiangnan.edu.cn (X.Z.); 2 Key Laboratory of Industrial Biotechnology of Ministry of Education, School of Biotechnology, Jiangnan University, Wuxi 214122, China; 3 China Key Laboratory of Microbiomics and Eco-Brewing Technology for Light Industry, Jiangnan University, Wuxi 214122, China; 4 Technical Research Institute, Shede Spirits Co., Ltd., Shehong 629000, China; lzguo@tuopai.biz (Z.L.); zyfang@tuopai.biz (Y.Z.); power@tuopai.biz (J.P.)

**Keywords:** *Baijiu*, linoleic acid oxides, aging markers, LC-MS/MS

## Abstract

**Background**: Post-production storage plays a pivotal role in developing the characteristic flavor profile of *Baijiu*, a traditional alcoholic beverage in China. While aging markers remain crucial for quality authentication, the identification of reliable metabolic indicators for chronological determination requires further exploration. **Methods**: This study establishes a novel liquid chromatography-tandem mass spectrometry (LC-MS/MS) methodology for quantifying five linoleic acid-derived oxidative metabolites in *Baijiu*: 9,12,13-trihydroxy-10(E)-octadecenoic acid (9,12,13-TriHOME), 9,10-Dihydroxy-12-octadecenoic acid (9,10-DiHOME), 9-oxo-(10E,12Z)-octadecadienoic acid (9-OxoODE), 9-hydroxy-(10E,12Z)-octadecadienoic acid (9-HODE) and 13-hydroxyoctadeca-(9Z,11E)-octadecadienoic acid (13-HODE). **Results**: The optimized protocol demonstrated exceptional sensitivity with limits of detection at 0.4 ppb through membrane-filtered direct dilution. Calibration curves exhibited excellent linearity (R^2^ > 0.9990) across 1.0–100.0 ppb ranges. Method validation revealed satisfactory recovery rates (87.25–119.44%) at three spiking levels (10/20/50 ppb) with precision below 6.96% RSD. Application to authentic samples showed distinct temporal accumulation patterns. Light-aroma *Baijiu* exhibited storage duration-dependent increases in all five oxides. Strong aroma variants demonstrated significant positive correlations for 9,12,13-TriHOME, 9,10-DiHOME, and 9-OxoODE with aging time. **Conclusions**: These findings systematically characterize linoleic acid oxidation products as potential aging markers, providing both methodological advancements and new insights into *Baijiu* aging mechanisms.

## 1. Introduction

The storage constitutes a pivotal phase for the production of distilled spirits, such as brandy [1], whiskey [2,3], and rum [4]. This process facilitates the formation of complex flavor compounds through the gradual occurrence of chemical reactions [5,6,7,8]. Additionally, a portion of the flavor compounds undergo extraction from oak into the spirits during this phase [9,10]. Thus, the storage process has been demonstrated to not only enhance the flavor profile of distilled spirits but also to improve their taste [8]. Therefore, the quality and value of distilled spirits are closely related to their maturation time. The development of methods for discriminating distilled spirits of different ages is of considerable interest to both researchers and manufacturers.

Volatile substances have been widely reported as potential aging markers for distilled spirits. For example, furfural, lactones, and acetals have been identified as potential age-related markers in Madeira wine [11]. In addition, furfural and its derivatives, hydroxymethylfurfural, were reported to be the most distinguishable age-related compounds for Brazilian rum [12]. In a recent study by Hu et al., oak-derived compounds, such as furfural, 5-methylfurfural, 4-ethylphenol in brandy, as well as β-damascenone, naphthalene, styrene, and decanal, were found to be positively correlated to aging time [13]. Zhu et al. identified 1,1-diethoxymethane and methanethiol as effective biomarkers of aging in Chinese roasted-sesame-like aroma and flavor-type liquor [14]. Wang et al. indicated that terpenoids and norisoprenoids can be used as aging markers for Qingke *Baijiu* [15]. Collectively, these studies suggested that the volatile compounds not only affect the aroma and flavor of distilled spirits, but also can serve as potential age-related markers.

However, with the mounting focus on the study of aging markers in distilled spirits, a contradiction emerges concerning the utilization of volatile components as age-related markers. For instance, Zhu et al. observed an increase in the content of ethyl ester in traditional Laowuzeng *Baijiu* during storage [16], while another study documented a decrease [17]. Furthermore, the volatile components of distilled spirits produced in disparate years, particularly trace components, are influenced by numerous factors, including raw materials, environmental conditions, and brewing bacteria [18]. This may result in inconsistent content, thereby constraining the utilization of volatile substances as aging markers.

A growing body of research has revealed that distilled spirits contain not only volatile compounds, but also numerous non-volatile compounds, especially *Baijiu* [19]. *Baijiu* is a traditional fermented alcoholic beverage in China. It is known as the world’s six most prominent distilled spirits, along with whisky, brandy, vodka, rum, and gin. The history of *Baijiu* extends hundreds of years, intricately intertwined with Chinese culture, serving as an integral component of social, ceremonial, and culinary traditions. It is made from various grains such as sorghum, rice, wheat, corn, or a combination of these grains by a complex multi-stain and solid-state fermentation process. Due to the different production processes and raw materials used, the final product may exhibit various aroma and flavor characteristics and can be classified into multiple categories, including strong-aroma, light-aroma, soy sauce-aroma, and rice-aroma *Baijiu*, etc. Among these, strong-aroma *Baijiu* and light-aroma *Baijiu* are the two most popular and widely consumed varieties. Typically, the saccharification and fermentation agents used for the production of strong-aroma and light-aroma *Baijiu* are *Qu*, functioning as a kind of equivalent to Japanese koji. The strong-aroma *Baijiu* is fermented in a pit covered with clay and characterized by a mellow taste. In contrast, the light-aroma *Baijiu* is usually fermented in an earthen jar and has a clean and refreshing taste. The selection of both strong-aroma and light-aroma *Baijiu* in this study allows us to explore the effect of different fermentation processes and flavor profiles on the formation of linoleic acid oxides during maturation.

The maturation process is of paramount importance in the production of *Baijiu*. The freshly distilled *Baijiu* typically undergoes a prolonged aging process, often spanning months or even years, prior to its bottling and commercialization [20]. Some premium *Baijiu* undergoes an extended maturation period, sometimes exceeding decades [21]. During the maturation process, a trace amount of oxygen perpetually enters a pottery jar, a conventional storage container for *Baijiu*. Consequently, the oxidation reaction transpires during the storage of *Baijiu*. The introduction of oxygen or the removal of hydrogen from organic compounds results in the production of novel trace substances [7,22]. However, the existing study on the understanding of the natural aging of *Baijiu* mainly focuses on the oxidation process of volatile components such as alcohol, phenol, and aldehyde [5], with comparatively limited involvement in the oxidation process of nonvolatile components. Therefore, further in-depth research is warranted to investigate the potential of using non-volatile compounds as aging markers.

Linoleic acid is classified as an omega-6 series polyunsaturated fatty acid, which has been identified in various types of *Baijiu* [23,24]. Linoleic acid in *Baijiu* is mainly derived from brewing raw materials (such as sorghum, wheat, and corn) and the metabolites of microorganisms during fermentation [25]. Despite the low content of linoleic acid in *Baijiu*, its chemical activity renders it susceptible to oxidation reactions. Upon the oxidation, linoleic acid can generate a variety of products, including 9,12,13-trihydroxy-10(E)-octadecenoic acid (9,12,13-TriHOME), 9-oxo-(10E,12Z)-octadecadienoic acid (9-OxoODE), 9-hydroxy-(10E,12Z)-octadecadienoic acid (9-HODE), 13-hydroxyoctadeca-(9Z,11E)-octadecadienoic acid (13-HODE) and 9,10-Dihydroxy-12-octadecenoic acid (9,10-DiHOME) [26,27]. During the aging process, these oxidation products may accumulate and thus have a certain relationship with storage time.

Thus, the objective of this study was to develop a method for the identification of linoleic acid oxidation products in *Baijiu* and to ascertain the potential utilization of these oxidation products as aging markers. To our knowledge, this is the first study attempting to use non-volatile components as age-related markers for distilled spirits. The study improved our understanding of the chemical composition of *Baijiu* and provided a new perspective for the quality evaluation and vintage identification of distilled spirits.

## 2. Materials and Methods

### 2.1. Baijiu Samples

Strong-aroma *Baijiu* samples of 0, 3, 5, 10, and 20 years were provided by a distillery in Sichuan province (30°30′35.46″ N, 105°34′33.13″ E). Light-aroma *Baijiu* samples of 5, 9, 14, 19, 20, 22, 23, 26 and 49 years were provided by a distillery in Shanxi province (37°20′19.98″ N, 111°54′03.96″ E).

### 2.2. Reagents and Standards

Chromatographic grade ethanol, acetonitrile, and methanol were purchased from Sinopharm Group Chemical Reagent Co., Ltd. (Shanghai, China). The experimental water is ultrapure water from the Milli-Q purification system (18.2 MΩ · cm).

Oxidation products of linoleic acid were purchased from Alta Technology Co., Ltd. (Tianjin, China). The detailed information regarding these compounds, including their abbreviations, CAS numbers, and chemical structures, is listed in Table 1.

### 2.3. Instruments and Equipment

QTRAP5500 liquid chromatography-triple quadrupole composite linear ion trap mass spectrometry, C8 liquid chromatography column (3 μm, 2.1 mm × 100 mm) and C18 liquid chromatography column (3 μm, 2.1 mm × 100 mm) American AB SCIEX Company (American Applied Biological Systems Company, Boston, MA, USA); mini vortex mixer Beijing Ranjeck Technology Co., Ltd. (Beijing, China); Concentrator Eppendorf Co., Ltd. (Hamburg, Germany); Millipore Synergy UV ultrapure water machine Millipore Company (Bedford, MA, USA).

### 2.4. Sample Extraction

Direct dilution method: 1 mL *Baijiu* sample was transferred into a 5 mL volumetric flask and diluted to the scale with absolute ethanol. The mixture was then vortexed for 2 min, filtered through a 0.22 μm organic membrane, and stored at 4 °C for analysis.

Vacuum concentration method: 2 mL of *Baijiu* sample was transferred to a centrifuge tube and concentrated in both alcohol-based (V-AL) and aqueous (V-AQ) modes. Then, 500 µL of methanol was added to the dried sample, followed by vortex for 1 min and centrifuge at 12,000 rpm at 4 °C for 10 min. The supernatants were then transferred to a new 2 mL tube and reconcentrated. The residues were re-dissolved in 150 µL of 80% methanol-water solution and filtered through a 0.22 μm membrane into a vial for analysis.

### 2.5. Preparation of Standard Solution

Accurately measure an appropriate amount of 5 linoleic acid oxide standard solution in a 10 mL capacity bottle, set the volume with ethanol absolute to the scale, make a 1 mg/L standard intermediate solution, and store it at −20 °C for use. Then, take a certain concentration of the prepared standard intermediate solution, prepare 1.0, 2.0, 5.0, 10.0, 20.0, 50.0, 100.0 μg/L standard working series solution, pass through the membrane, and wait for analysis.

### 2.6. Instrument Parameters

Using LC-MS/MS to establish a quantitative analysis method of 5 linoleic acid oxides through multi-reaction monitoring (MRM) mode [28]. The mass spectrometry parameters are as follows: scanning mode is ESI negative ion mode, ion source temperature 120 °C, capillary voltage 30 V, cone voltage 30 V. Nitrogen was used as cone gas (with flow rate 50 L/h) and desolvation gas (with flow rate of 40 L/h at 350 °C). Argon was used as the collision gas at a flow rate of 0.2 mL/min to induce fragmentation for MS/MS analysis.

**Figure 1:**
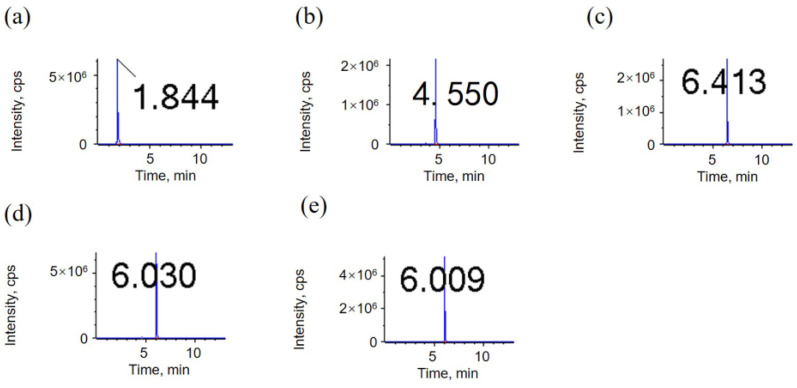
LC–MS/MS chromatograms of 1000 μg/L of 5 linoleic acid oxide standards in the MRM mode; (**a**) 9,12,13-trihydroxy-10(E)-octadecenoic acid (THO); (**b**) 9,10-Dihydroxy-12-octadecenoic acid (DHO); (**c**) 9-oxo-(10E,12Z)-octadecadienoic acid (OXO); (**d**) 9-hydroxy-(10E,12Z)-octadecadienoic acid (9HO); (**e**) 13-hydroxyoctadeca-(9Z,11E)-octadecadienoic acid (13HO). The numbers in the graph represent the retention time (RT).

Chromatography parameters: the water phase of the mobile phase A is 0.1% formic acid solution, the organic phase of the mobile phase B is acetonitrile, the column temperature is 50 °C, the sample volume is 2 μL, and the flow rate is 0.35 mL/min. The elution conditions are: 0 min, A/B (60:40); 1 min, A/B (60:40); 7 min, A/B (30:70); 8 min, A/B (1:99); 10 min, A/B (1:99); 10.1 min, A/B (60:40); 13 min, A/B (60:40). The mass spectrometry parameters and chromatographic peak of the oxides to be measured are shown in Table 2 and Figure 1 below.

### 2.7. Method Validation

Validate the established quantitation methods, including linearity, limit of detection (LOD), limit of quantification (LOQ), recovery, and precision (RSD, %).

#### 2.7.1. Linearity, LOD, and LOQ

This experiment adopts the external standard method for quantitative analysis [29,30,31]. In the concentration range of 1–100 μg/L (ppb), the linearity of each analyte was evaluated by establishing a calibration curve. Least squares linear regression was used to calculate the slope, intercept, and R^2^ values. A correlation coefficient exceeding 0.99 was considered desirable for all calibration curves.

The LODs were calculated from the lowest concentration of the calibration curve at a signal-to-noise ratio of 3 (S/N = 3) and the LOQs were determined at the lowest concentration of the concentration at a signal-to-noise ratio of 10 (S/N = 10) [32]. All analyses were performed in 6 replicates (n = 6).

#### 2.7.2. Accuracy and Precision

Using 60% ethanol as a matrix, 5 linoleic acid oxide standard solutions of known concentrations were added. Recovery of 5 linoleic acid oxides was substantiated through recovery trials conducted at three distinct concentration levels (10, 20, and 50 ppb). Recovery is calculated as recovery (%) = (measured value after sample addition—measured value before sample addition) × 100%/sample addition. Six replicates were performed for each sample, and the results were considered acceptable in the range of 80% to 120%.

Precision was expressed in the form of the relative standard deviation (RSD%) of each spiking level. Both intra-day and inter-day precision showed reproducibility. Intra-day precision was investigated by analyzing 6 replicates of samples on the same day. Inter-day precision was assessed by analyzing three samples over three consecutive days. RSD is calculated as RSD (%) = Standard Deviation (SD)/Arithmetic Mean of the Calculated Results (X) × 100%.

### 2.8. Data Analysis

LC-MS/MS Analyst software combined with SCIEX OS 1.4.0.18067 (SCIEX, Toronto, QC, Canada) qualitative and quantitative analysis software to process the experimental data, and Microsoft Excel 2021 was used for preliminary statistical analysis. Linear regression analysis was performed using GraphPad Prism 8.0.1 software combined with least squares method and graphed.

## 3. Results

### 3.1. Optimization of Detection Conditions

#### 3.1.1. LC Column Selection

C8 and C18 columns are commonly used chromatographic columns in fatty acid analysis due to their good compatibility [33,34,35,36]. While both columns generate a stationary phase by alkyl bonding on the silica gel surface, and the mobile phase usually remains aqueous and moderately polar, the C8 column uses octyl carbon chain-bonded silica gel with 8 carbon atoms as the stationary phase, which has low hydrophobicity, and the C18 column uses octadecyl carbon chain-bonded silica gel, which has high hydrophobicity. Both columns are employed for the analysis of weakly polar substances, with C8 being suitable for substances with slightly higher polarity and C18 being suitable for substances with weaker polarity. The objective was to ascertain which column provided the optimal separation. To select an optimal column for the separation of five linoleic acid oxides, we evaluated the performance of the C8 and C18 columns.

**Figure 2:**
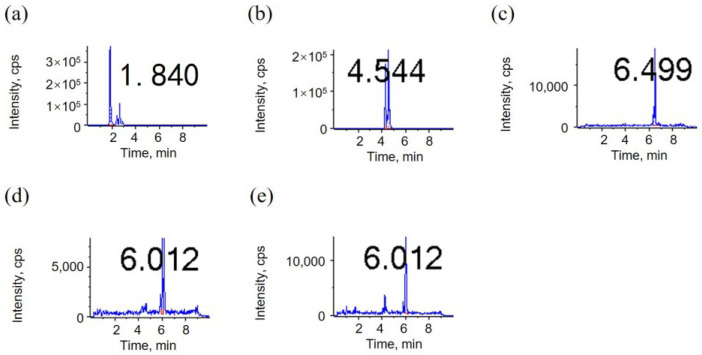
LC–MS/MS chromatograms of 5 linoleic acid oxides on C8 column in the MRM mode; (**a**) THO; (**b**) DHO; (**c**) OXO; (**d**) 9HO; (**e**) 13HO. The numbers in the graph represent the RT.

**Figure 3:**
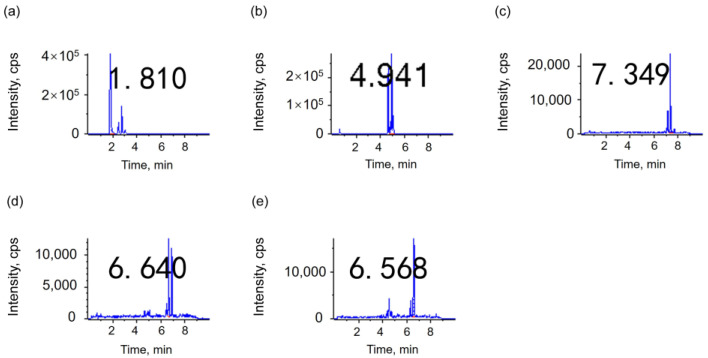
LC–MS/MS chromatograms of 5 linoleic acid oxides on C18 column in the MRM mode; (**a**) THO; (**b**) DHO; (**c**) OXO; (**d**) 9HO; (**e**) 13HO. The numbers in the graph represent the RT.

Although the peak area response of the C8 (Figure 2) and C18 (Figure 3) columns is on an order of magnitude, the C8 column has a better separation (Figure 2). The C18 column exhibits higher retention than the C8 column, rendering it more suitable for the analysis of macromolecules. In comparison to saturated fatty acids, linoleic acid oxides contain multiple polar functional groups, resulting in enhanced polarity. This heightened polarity of linoleic acid oxides may also be a contributing factor to the superior separation of linoleic acid oxides observed in the C8 column [37]. From the perspective of the peak time obtained by instrumental analysis, Table 3 shows that the peak time of the C18 column is later than that of the C8 column, which is consistent with the column properties. Collectively, the C8 column is more suitable as a liquid chromatography column for substance detection.

#### 3.1.2. Optimization of Pretreatment Method

**Figure 4:**
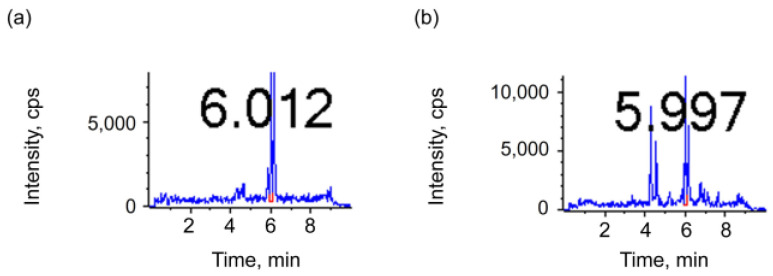
LC–MS/MS chromatograms of 9HO in different pretreatments on C8 column in the MRM mode. (**a**) Direct dilution pretreatment; (**b**) Vacuum concentration. The numbers in the graph represent the RT.

Direct dilution and vacuum concentration are two commonly used methods in sample processing. The direct dilution method reduces the concentration of the target substance and improves the detection sensitivity by adding a solvent to the sample. The vacuum concentration method improves the detection sensitivity by removing the solvent by evaporation under reduced pressure. In the present study, ethanol was selected for direct dilution in the *Baijiu* samples due to its role as the predominant component and its ability to avoid the introduction of extraneous substances. Concurrently, the *Baijiu* samples underwent vacuum concentration in V-AL and V-AQ modes [38,39]. As is shown in Table 4, the content of the five oxides is higher under direct dilution than under vacuum concentration, and the loss due to direct dilution is less. Moreover, the oxides exhibit a reduced number of peaks and are less susceptible to interference under direct dilution (Figure 4).

### 3.2. Linear Range, Detection Limit (LOD), and Quantitative Limit (LOQ)

Under the aforementioned conditions, a series of standard working solutions of 1.0, 2.0, 5.0, 10.0, 20.0, 50.0, and 100.0 ppb were prepared and analyzed on the machine. The calibration curve and determination correlation coefficients (R^2^) for the five linoleic oxides are presented in Table 5. The method showed good linearity for these linoleic oxides in the calibration range, with R^2^ > 0.9990. All of the linoleic oxides in this study had LODs of 0.4 ppb and LOQs of 1 ppb. The LODs and LOQs were lower than previously reported for 5 linoleic oxides in *Baijiu* samples [27]. However, THO exhibited higher LOD and LOQ compared to a prior trial, which were 0.2 ppb and 0.5 ppb [40], respectively, and may be attributed to the fact that a single target substance is the sole object of detection and the detection signal is derived from it alone. The results of LOD, LOQ, and the linear range indicate that the proposed method demonstrates adequate sensitivity and precision.

### 3.3. Recovery and Intra-Day and Inter-Day Precision

Table 6 shows the accuracy and precision of the developed method for the analysis of five linoleic acid oxides. The recovery, intra-day RSD, and inter-day RSD of this method were verified at low (10 ppb), middle (20 ppb), and high (50 ppb) spiking levels. Recoveries of the five linoleic acid oxides ranged from 80.51% to 119.92% at low concentrations, 100.51% to 119.44% at middle concentrations, and 105.02% to 118.14% at high concentrations. The range of RSD values for intra-day precision was from 0.55 to 6.66% (low-level), from 0.39 to 1.42% (middle level), and from 1.01 to 2.95% (high level). RSD values for inter-day precision ranged from 0.43 to 6.96% (low), 0.50 to 2.47% (middle), and 0.50 to 4.76% (high). In summary, the intra-day precision, inter-day precision, and relative standard deviation of all analytes were less than 7%, and the recoveries were within the range of 80.51–119.92%, which was within the acceptable range of this parameter, indicating that the experimental method had good reliability. Supporting these findings, a recent study utilizing ultra-performance liquid chromatography-high-resolution mass spectrometry (UPLC-HRMS) reported recoveries of THO at three spiked levels (5, 15, and 20 ng/mL) ranging from 83.40% to 103.55%, with RSD values between 2.14% and 6.89% [40]. This further confirms the reliability of the method. Similarly, another study employing UPLC-MS/MS for the determination of linoleic acid oxides in *Baijiu* achieved RSD values ranging from 2.2% to 8.9% and recoveries between 92.0% and 110.8% [27]. These results collectively underscore the accuracy, precision, and reproducibility of the analytical approach, highlighting its suitability for the quantification of linoleic acid oxides in complex matrices such as alcoholic beverages.

### 3.4. Determination of 5 Linoleic Acid Oxides in Strong-Aroma Baijiu with Different Storage Time

**Figure 5:**
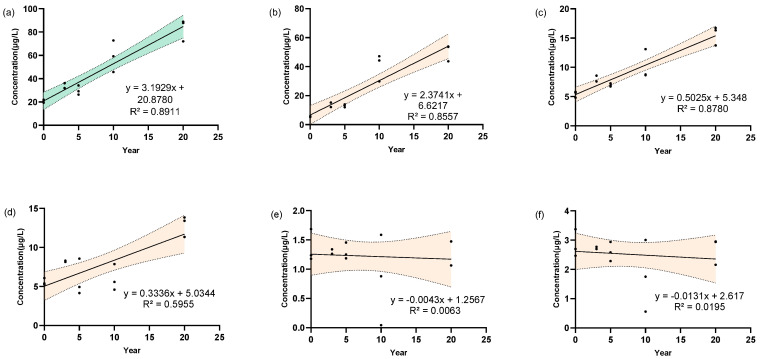
Linear regression of the linoleic acid oxides in strong-aroma *Baijiu* with storage time. Storage time vs. (**a**) Total concentration of 5 linoleic acid oxides; (**b**) THO; (**c**) DHO; (**d**) OXO; (**e**) 9HO; (**f**) 13HO linear regression with corresponding R^2^ value. The colored areas in the graph represent the 95% confidence interval for the regression curve.

In order to investigate the influence of storage time on the content of linoleic acid oxides in *Baijiu*, Strong-aroma *Baijiu* with different storage times, ranging from 0~20 years, were selected to determine their content. As is shown in Figure 5, linear fitting analysis (Figure 5a) demonstrated an R^2^ value of 0.8911 between the total content of the 5 oxides and the storage time. Furthermore, the R^2^ of THO (Figure 5b) and DHO (Figure 5c) content and storage time were 0.8557 and 0.8780, respectively. However, the R^2^ values for OXO were determined to be 0.5955 and the R^2^ values for 9HO and 13HO were found to be less than 0.1, as depicted in Figure 5d–f. Although the correlation is weak, it may reflect the degradation of oxides in *Baijiu*. The degree of oxidation of the sample can be determined by measuring the lipid peroxide value or comparing the microbial community differences (analyzed by qPCR or pyrosequencing). The weak correlations may also reflect nonlinear relationships or variability in storage conditions across samples. To better understand the formation of these compounds, future studies could investigate the effect of oxidative pathways and microbial metabolism on the production of these compounds. The execution of controlled aging experiments, encompassing the modulation of parameters such as oxygen exposure, microbial inoculation, and storage conditions, holds the potential to elucidate the underlying mechanistic drivers and optimize synthesis.

### 3.5. Determination of 5 Linoleic Acid Oxides in Light-Aroma Baijiu with Different Storage Time

**Figure 6:**
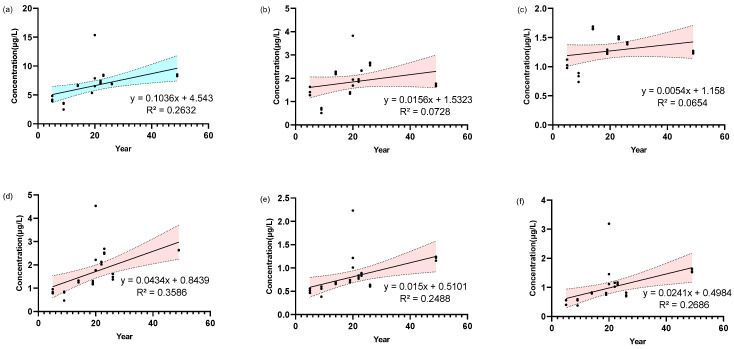
Linear regression of the linoleic acid oxides in light-aroma *Baijiu* with storage time. Storage time vs. (**a**) Total concentration of 5 linoleic acid oxides; (**b**) THO; (**c**) DHO; (**d**) OXO; (**e**) 9HO; (**f**) 13HO linear regression with corresponding R^2^ value. The colored areas in the graph represent the 95% confidence interval for the regression curve.

In the present study, the light-aroma *Baijiu* with different storage times ranging from 5 to 49 years were subjected to the aforementioned method of analysis (Figure 6). As demonstrated in Figure 6a, despite the variability identified between the concentration of linoleic oxides and storage time, a general trend can still be observed. The observed weak correlations may be attributed to the intricate formation pathways of linoleic acid oxides, coupled with their potential for subsequent chemical transformations or degradation during storage. These dynamic processes can result in fluctuations in their concentrations that deviate from a strictly linear trend. For example, certain oxides may reach a concentration plateau or undergo degradation over prolonged storage periods. These phenomena are not adequately captured by a linear regression model. Furthermore, variability in raw materials, fermentation protocols, and storage conditions (e.g., temperature, humidity, oxygen exposure) across samples may introduce heterogeneity into the data, thereby attenuating the strength of the observed correlations. Future studies can design storage experiments conducted under controlled conditions, such as constant temperature, constant humidity, and specific oxygen concentrations, to reduce sample-to-sample variability. Also, design time-series experiments with periodic sampling and analysis to more accurately track the dynamics of linoleic acid oxides.

## 4. Discussion and Conclusions

The detection of trace compounds has always been a challenge in the field of food research. This is particularly evident in the differentiation and quantification of structurally similar compounds. At present, high-sensitivity techniques such as GC-MS or LC-MS are commonly used to accurately quantify trace compounds in various food samples. Compared with LC-MS, GC-MS requires not only derivatization, but also purification steps to remove impurities formed during derivatization [41]. For the detection of oxides, LC-MS has shown its advantages [42]. The LC-MS/MS technique employed in this study utilizes secondary mass spectrometry, which not only improves sensitivity, but also effectively distinguishes isomers by fragment ions [43], achieving the distinction between 9HO and 13HO with high accuracy. In addition, the use of LC-MS/MS has enabled the precise quantification of 9HO and 13HO concentrations in mouse neutrophils [44], thereby substantiating the reliability and relevance of this analytical technique in the study of lipid metabolites. Significant advancements have been made in the field of food science with the application of LC-MS/MS technology. For instance, the dynamic accumulation of lipids oxidized by linoleic acid during the storage of hazelnut oil was revealed for the first time by HPLC-MS-targeted lipidomics in MRM mode [45]. This provided important data support for the study of the oxidation stability of oils. In other food matrices, hydroperoxyoctadecadienoic acid (HPODE), a linoleic acid oxidation product generated by soybean oil and olive oil, was recently qualitatively and quantitatively analyzed by LC-MS/MS [46]. Collectively, the based approach provides a powerful analytical method for both the detection and quality control of food oxidation products.

The present study established a rapid detection method for the content of 5 linoleic acid oxides in *Baijiu* by LC-MS/MS, and explored the changes in the content of these 5 oxides in strong-aroma and light-aroma *Baijiu* over different storage periods. The results indicated that the content of linoleic acid oxides exhibited a certain correlation with the storage time of *Baijiu*.

As metabolites of linoleic acid, linoleic acid oxides can be produced through enzymatic pathways in vivo or non-enzymatic pathways in vitro. It has been suggested that linoleic acid oxides have a wide range of biological functions [45,47,48]. Among the 5 linoleic acid oxides examined in the present study, THO has been identified as a promising adjuvant and anti-inflammatory agent [49]. Meanwhile, OXO was shown to enhance dyslipidemia management by modulating the activity of peroxisome proliferator-activated receptors (PPARs) [50]. Notably, 13HO has anti-inflammatory effects as peroxisome proliferator-activated receptor γ (PPAR γ) agonists and increases the biosynthesis of prostacyclin (PGI2) [51,52]. Nevertheless, DHO is a leukocyte toxin derivative of linoleate glycol, which is cytotoxic, and high concentrations lead to mitochondrial dysfunction, increased cellular oxidative stress, and apoptosis [53]. Notably, 9HO has been shown to be pro-inflammatory in experimental wound healing models in rats, which can promote the formation of atherosclerosis [52]. Therefore, linoleic acid oxides are not only potential aging markers for *Baijiu*, but also facilitate product quality control and provide consumers with a scientific foundation for understanding its health implications.

Although the present study has achieved a quantitative analysis of linoleic acid oxides in *Baijiu*, some limitations must be acknowledged. First, as illustrated in Figure 6, particularly in (a) to (c), the data for the 49-year-old *Baijiu* seems to be different from the other samples. After excluding the data from the 49-year-old *Baijiu* samples, the R^2^ for the total linoleic acid oxides improved from 0.2632 to 0.3412. Similarly, the R^2^ values for THO and DHO increased significantly from 0.0728 to 0.3830 and from 0.0654 to 0.3697, respectively, indicating a stronger correlation with storage time. However, the R^2^ values for 9-HODE and 13-HODE exhibited a decline, from 0.2488 to 0.1296 and from 0.2686 to 0.1738, respectively. These results indicate that the 49-year-old sample may represent an outlier or a unique case, possibly due to extended storage conditions leading to further oxidation or degradation of some linoleic acid oxides. For example, compounds such as 9-HODE and 13-HODE may undergo additional chemical reactions over time, resulting in the transformation into other products. Further studies are needed to explore these possibilities and to better understand the long-term behavior of linoleic acid oxides during *Baijiu* aging. Furthermore, the variability of linolenic acid oxides in other aroma types of *Baijiu*, such as say sauce-aroma or rice-aroma *Baijiu*, remains unclear. To further elucidate this relationship, it is necessary to expand the sample size to include different aroma types of *Baijiu*. In addition, due to the complex composition and diverse oxidation pathways of *Baijiu*, the current research has not elucidated the oxidation mechanism of linoleic acid. Future research should integrate metabolomics, enzymology, and molecular biology technologies to explore the formation pathways and regulatory mechanisms of linoleic acid oxides in *Baijiu*.

## Figures and Tables

**Table 1:** Key information of 5 linoleic acid oxides.

Compounds	Abbreviation	Short Abbreviation	CAS Number	Chemical Structure
9,12,13-trihydroxy-10(E)-octadecenoic acid	9,12,13-TriHOME	THO	97134-11-7	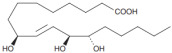
9,10-Dihydroxy-12-octadecenoic acid	9,10-DiHOME	DHO	263399-34-4	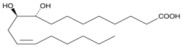
9-oxo-(10E,12Z)-octadecadienoic acid	9-OxoODE	OXO	54232-59-6	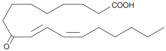
9-hydroxy-(10E,12Z)-octadecadienoic acid	9-HODE	9HO	98524-19-7	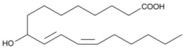
13-hydroxyoctadeca-(9Z,11E)-octadecadienoic acid	13-HODE	13HO	73804-64-5	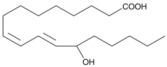

**Table 2:** Mass spectrometric parameters of 5 linoleic acid oxides.

Compounds	Ionization Mode	Q1 ^a^	Q3 ^b^	Ion ratio	Dwell Time	DP ^c^	CE ^d^
THO	Negative	329.2	211	1.41	1.79	−120.000	−31.000
DHO	Negative	313.2	171	1.18	4.48	−120.000	−31.000
OXO	Negative	293.2	185	2.81	6.36	−120.000	−27.000
9HO	Negative	295.2	171	1.71	5.97	−120.000	−24.000
13HO	Negative	295.2	195	2.08	5.95	−120.000	−26.000

^a^ Q1, parent ion. ^b^ Q3, fragment ion. ^c^ DP, declustering potential. ^d^ CE, collision energy.

**Table 3:** RT (min) of 5 linoleic acid oxides in different columns.

Columns	THO	DHO	OXO	9HO	13HO
C8	1.84	4.63	6.50	6.01	6.01
C18	1.81	5.04	7.35	6.64	6.57

**Table 4:** Contents of 5 linoleic acid oxides in different pretreatments on C8 column in the MRM mode (ppb).

Pretreatments	THO	DHO	OXO	9HO	13HO
Directly dilute	210.15	242.65	27.935	1.521	11.735
Vacuum concentration	175.15	53.65	17.215	0.2583	1.4785

**Table 5:** Linear range, calibration curve, determination coefficient, LOD, and LOQ data of 5 linoleic acid oxides in spiked samples (n = 6).

Compounds	Linear Range (ppb)	Calibration Curve	R^2 a^	LOD ^b^ (ppb)	LOQ ^c^ (ppb)
THO	1–100	y = 40,411.6x + 13,072.6	0.9995	0.4	1.0
DHO	1–100	y = 77,417.3x + 15,682.2	0.9994	0.4	1.0
OXO	1–100	y = 11,560.3x + 7910.8	0.9995	0.4	1.0
9HO	1–100	y = 31,758.4x + 25,882.0	0.9990	0.4	1.0
13HO	1–100	y = 20,198.3x + 19,326.5	0.9993	0.4	1.0

^a^ R^2^, coefficient of determination. ^b^ LOD, limit of detection. ^c^ LOQ, limit of quantification.

**Table 6:** Recoveries and precision of 5 linoleic acid oxides in spiked samples (n = 6).

Compounds	Recovery (%)			Average Recovery (%)			Intra-Day RSD ^d^ (%)			Inter-Day RSD (%)		
	Low ^a^	Middle ^b^	High ^c^	Low	Middle	High	Low	Middle	High	Low	Middle	High
THO	88.13~92.70	105.09~108.88	105.05~107.86	90.12	106.91	106.71	2.01	1.42	1.01	3.60	2.47	1.04
DHO	97.93~118.63	112.23~117.21	109.95~117.85	111.91	115.03	115.14	6.66	1.42	2.72	6.96	2.13	0.50
OXO	80.51~85.32	100.51~107.85	107.43~118.14	83.19	104.49	112.89	0.55	1.24	2.95	0.63	1.71	0.90
9HO	108.07~119.92	106.81~109.73	105.02~109.96	115.01	108.29	108.16	3.00	0.89	1.46	4.03	0.45	0.82
13HO	94.03~97.93	118.29~119.44	108.11~112.38	95.60	118.79	110.39	1.65	0.39	1.77	6.75	0.43	4.76

^a^ Low, low spiked levels (10 ppb). ^b^ Middle, middle spiked levels (20 ppb). ^c^ High, high spiked levels (50 ppb). ^d^ RSD, relative standard deviation.

## Data Availability

The datasets are available from the corresponding author upon reasonable request.

## Abbreviations

The following abbreviations are used in this manuscript:

LC-MS/MS	Liquid Chromatography-Tandem Mass Spectrometry
LC-MS	Liquid Chromatograph Mass Spectrometer
GC-MS	Gas Chromatography Mass Spectrometry
THO	9,12,13-trihydroxy-10(E)-octadecenoic acid
DHO	9,10-Dihydroxy-12-octadecenoic acid
OXO	9-oxo-(10E,12Z)-octadecadienoic acid
9HO	9-hydroxy-(10E,12Z)-octadecadienoic acid
13HO	13-hydroxyoctadeca-(9Z,11E)-octadecadienoic acid
MRM	Multi Reaction Monitoring
RT	Retention Time
LOD	Limit of Detection
LOQ	Limit of Quantification
RSD	Relative Standard Deviation
SD	Standard Deviation
R^2^	Coefficient of Determination
PPARs	Peroxisome Proliferator-Activated Receptors
PPARγ	Peroxisome Proliferator-Activated Receptors γ
PGI2	Prostacyclin
